# Pneumoperitoneum with Subcutaneous Emphysema after Percutaneous Endoscopic Gastrostomy

**DOI:** 10.1155/2014/726878

**Published:** 2014-07-10

**Authors:** Yalin Iscan, Bora Karip, Yetkin Ozcabi, Birol Ağca, Yesim Alahdab, Kemal Memisoglu

**Affiliations:** ^1^General Surgery, Fatih Sultan Mehmet Eğitim ve Araştırma Hastanesi, İçerenköy, 34752 İstanbul, Turkey; ^2^Gastroenterology, Fatih Sultan Mehmet Eğitim ve Araştırma Hastanesi, İçerenköy, 34752 İstanbul, Turkey

## Abstract

Percutaneous endoscopic gastrostomy is a safe way for enteral nutrition in selected patients. Generally, complications of this procedure are very rare but due to patients general health condition, delayed diagnosis and treatment of complications can be life threatening. In this study, we present a PEG-related massive pneumoperitoneum and subcutaneous emphysema in a patient with neuro-Behçet.

## 1. Introduction

Percutaneous endoscopic gastrostomy (PEG) has become the most preferred procedure for long-term enteral feeding since its description in the early 1980s [1]. It was reported as the second leading indication for upper gastrointestinal tract endoscopy in the USA [2]. PEG is proven to be safe, cost effective, and feasible. Most of the complications after PEG procedure are clinically minor and the frequency of serious complications is very low. A meta-analysis reported PEG-related morbidity of 9.4% and mortality of 0.53% [3]. With a neuromuscular disease or in a sedated patient, the diagnosis of complications may be delayed.

High clinical suspicion and early screening methods are essential for diagnosis, appropriate treatment, and favourable outcome [4]. In this study, we present a PEG-related massive pneumoperitoneum and subcutaneus emphysema in a patient with neuro-Behçet.

## 2. Case Presentation

**Figure 1:**
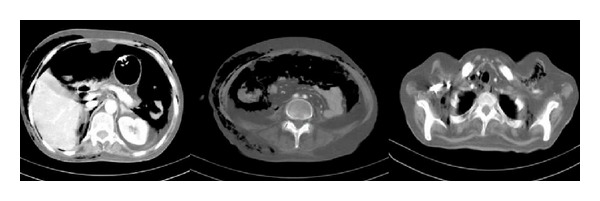
Thoracoabdominal CT, showing detached gastric wall from catheter insertion site and massive subcutaneous emphysema through cervical, thoracic, and abdominal region.

A 45-year-old woman, who was diagnosed as having Neuro-Behçet's disease (NBD), was admitted to our hospital with fever and cough. A PEG was performed 15 days ago due to the swallow dysfunction and standard enteral nutrition was applied after the procedure without any complaint. Her physical examination revealed a subcutaneous emphysema but there was no sign of peritoneal inflammation and symptoms of acute abdomen due to her neuromuscular disease. Also there was no wound infection around the PEG-tube. Her fever was 38,3 C. The hemoglobin concentration was 9.5 g/dL (12–16 gr/dL), leukocyte count was 9.8 K/uL (4–10 K/uL), platelet count was 215 K/uL (150–450 K/uL), and C-reactive protein level was 6.4 mg/dL (0-1 mg/dL). Liver and renal function tests were normal. Thoracoabdominal computerized tomography (CT) showed the presence of pneumoperitoneum with subcutaneous emphysema over the abdomen wall extending to the cervical and lomber region. The PEG tube was in the stomach in CT but the gastric wall was not attached to the abdominal wall (Figure 1).

**Figure 2:**
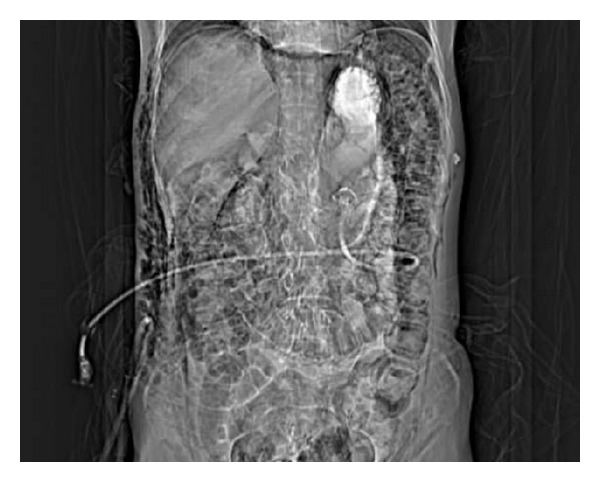
Abdominal X ray with contrast given from the PEG catheter. There was no intra-abdominal leakage.

After pulling up the PEG-tube and fixing the gastric wall to the abdominal wall, we checked the tube's position by a plain abdominal graph with contrast given through PEG catheter and there was no extra luminal contrast leakage (Figure 2).

**Figure 3:**
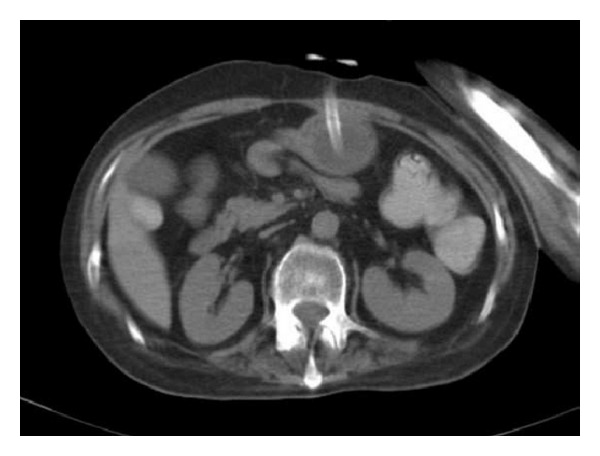
Replaced PEG catheter in the stomach and resolved pneumoperitoneum.

Enteral feeding was stopped and intravenous hyperalimentation was given after repositioning of the tube. Because of the fever and high levels of CRP, blood, urine, and deep tracheal aspiration (DTA) cultures were obtained. The culture results of DTA were positive for Klebsiella pneumonia so Ertapenem therapy was ordered according to the antibiogram results. The subcutaneous emphysema resolved within 7 days. We started enteral nutrition again seven days after admission, but there was a leakage back to the skin around the PEG tube. We replaced the PEG-tube with a 20-F foley catheter and fixed it to the skin with sutures. After this replacement, tube feeding was resumed successfully for seven days. On the fourteenth day of her admission, a new PEG catheter was inserted from another part of the stomach and the transient foley catheter was removed. No recurrence of leakage, pneumoperitoneum, and emphysema developed (Figure 3). She was discharged seventeen days after her admission.

## 3. Discussion

PEG is the second leading indication for upper gastrointestinal endoscopy in the US [2–5]. The number of patients with PEG tubes has increased significantly and will continue to increase. It is clear that these high volumes will bring higher numbers of complications. Morbidity rates of the procedure range from 9% to 17% but major complications are under 5% and mortality is lower than 1% [6, 7]. Papakonstantinou et al. divided the complications into three subgroups [8] (Table 1).

Pneumoperitoneum is common after PEG procedure, with an incidence of over 50% [9–11]. Probably the etiology of pneumoperitoneum or leakage occurs by insufficient fixation of the PEG, causing leakage of air through the gastric wall which enters the free peritoneal space. It is also explainable that air escapes through the small opening from the stomach during the interval between the initial needle puncture and the PEG tube passage through the abdominal wall. In the absence of symptoms with patients who have undergone a recent PEG, conservative management in pneumoperitoneum is suggested. Pneumoperitoneum is usually subclinical and self-limiting and should be clinically concerned only when intra-abdominal air is worsening or when it is found in the presence of signs of peritonitis, portal and/or mesenteric venous gas, systemic inflammatory response, and/or sepsis [11].

Subcutaneous emphysema is a very rare complication of PEG [12]. Emphysema was also described after percutaneous gastrostomy in which the catheter was placed under ultrasonography guidance with the asistance of fluoroscopy. It was reported that the evidence of subcutaneous emphysema occured after the fourth day of the procedure [11]. For our patient it was more than two weeks from the PEG insertion to diagnosis of emphysema. And also the patient's complaint was cough and fever which were not specific to emphysema.

Subcutaneous emphysema with gastrointestinal origin is very rare. Peptic ulcer perforation, trauma, carcinoma, diverticulitis, appendicitis, jejunal perforation, colonoscopy, dental surgery, and some acetebular orthopedic surgeries should be kept in mind [13, 14]. For our patients, it was due to the detachment of the gastric wall from abdominal wall.

There is no gold standard for treatment leakage and pneumoperitoneum after PEG. PEG leakage is reported by 58%–78% of patients with long-term PEG tube placement [15]. Leakage from the stoma occurs because of the dilatation of the stoma [16]. Removing the tube for a few days reduces the diameter of the stoma and permits a resized tube replacement [17].

The PEG tract closes in 24–48 hours when the patient is treated with bowel rest with or without nasogastric suction. Subsequent placement of a PEG tube in a new site is often successful. Repositioning or gastric wall fixation by another tube will not always stop the leakage in the same site because all stomas diameters do not reduce always by different manuplations.

Sometimes only repositioning of the catheter is enough. With our patient, although we checked the catheter with X-ray by infusing contrast from the catheter and confirmed it was placed correctly, we faced a leakage problem. An alternative way to solve leakage is the replacement of the PEG tube with a balon catheter or foley catheter. Foley catheters should only be used as temporary replacements to maintain the integrity of the fistula. In addition, the catheters should be marked in some way to determine the depth of insertion prior to inflation. Clinicians must pay attention to fix these kinds of catheters to the skin because of the catheter migration risk. By the propulsive force of gastric peristaltism, the head of tube may lead to a mechanical obstruction through duodenum and this may also cause pancreatitis [18]. If there is any doubt as to the location of any replacement tube, the position of the tube should be confirmed radiographically before inflation and the resumption of tube feedings.

In summary, the number of patients with PEG tubes has increased significantly and will continue to increase. An increased awareness of these rare but potentially life threatening complications is important. In this critically ill, comatose patient group, missing possible but rare complications may be lethal.

## Figures and Tables

**Table 1:** Complications of PEG procedure.

Due to the endoscopy procedure	Due to the PEG and the gastrostomy tube	Due to the mode of feeding
(i) Laryngospasm, airway obstruction (ii) Aspiration and pneumonia (iii) Respiratory depression or apnea (iv) Desaturation or respiratory distress and acute respiratory failure (v) Hypertension (vi) Fracture of the alveolar ridge while attempting to open the mouth	(i) Perforation/laceration of the oesophagus or the stomach (ii) Transhepatic insertion of the tube (iii) Pneumoperitoneum (iv) Colonic perforation (v) Subcutaneous emphysema (vi) Retroperitoneal hemorrhage (vii) Aortic perforation (viii) Erosion of the gastric mucosa and bleeding (ix) Hematoma or infection of the abdominal wall (x) Gastrocolic fistula (xi) Colocutaneous fistula (xii) Hypertrophic granulation tissue at the gastrostomy exit (xiii) Buried bumper syndrome (xiv) Malpositioning of the tube or leakage (a) To the subcutaneous tissues → cellulitis, myositis, necrotizing fasciitis, subcutaneous abscess. (b) To the peritoneal cavity → peritonitis, intraabdominal abscess, sepsis. (xv) Migration of the tip of the gastrostomy tube (a) To oesophagus (oesophagitis) (b) To pylorus (obstruction or perforation of the duodenum) (xvi) Migration of the whole PEG tube up to the terminal ileum (xvii) Peristomal hernia or stomal prolapse (xviii) Accidental pulling out or cutting off the tube close to the skin during home care (xix) Erosion of the tube through the gastric wall (xx) Obstruction of the tube lumen (xxi) Hub detachment or damage (xxii) Later symptomatic gastroesophageal reflux (xxiii) Ileus	(i) Diarrhoea (ii) Nausea (iii) Vomiting (iv) Dumping syndrome (v) Ogilvie's syndrome (vi) Aspiration pneumonia (vii) Constipation and meteorism
